# Socioeconomic inequalities in lung cancer – a time trend analysis with German health insurance data

**DOI:** 10.1186/s12889-021-10576-4

**Published:** 2021-03-19

**Authors:** Fabian Tetzlaff, Jelena Epping, Juliane Tetzlaff, Heiko Golpon, Siegfried Geyer

**Affiliations:** 1 Institute for General Practice, Hannover Medical School, Hanover, Germany; 2 Medical Sociology Unit, Hannover Medical School, Hanover, Germany; 3 Comprehensive Cancer Center Hannover, Hannover Medical School, Hanover, Germany; 4 Department of Pneumology, Hannover Medical School, Hanover, Germany

**Keywords:** Lung cancer, Time trend, Incidence, Socioeconomic inequalities, Germany

## Abstract

**Background:**

Lung Cancer (LC) is one of the most prevalent cancer diseases. Due to the lack of databases which allow the combination of information on individual socioeconomic status (SES) and cancer incidence, research on social inequalities in LC among the German population is rare. The aim of the study is to analyse time trends in social inequalities in LC in Germany.

**Methods:**

The analyses are based on data of a large statutory health insurance provider. The data contain information on diagnoses, occupation and education (working age), and income (full age range) of the insurance population. Trends were analysed for two subpopulations (retirement age and working age) and stratified by sex. The analyses are based on incidence rates and proportional hazard models spanning the periods 2006–2009, 2010–2013 and 2014–2017.

**Results:**

Incidence rates declined in men but increased in women. For men, inequalities were strongest in terms of income and the decline in incidence was most pronounced in middle- and higher-income men. Among women at retirement age, a reversed income gradient was found which disappeared in the second period. The educational gradient among the working-age population decreased over time due to the trend towards increasing incidence among individuals with higher education. Declining gradients were also found for occupational position.

**Conclusion:**

The findings reveal considerable inequalities in LC and that trends vary with respect to SES, sex and age. Widening income inequalities were found in the retired population, while educational and occupational inequalities tend to narrow among the working-age population.

## Background

Lung cancer (LC) is one of the most common cancer diseases and ranks among the most frequent causes of death in Germany [1, 2]. With regard to the development of incidence over time, different trends were observed for men and women. In the last decades, a decreasing number of incident cases was reported for men. In contrast, the yearly incidence in women rose continuously [2–4]. The strong link between socioeconomic status (SES) and morbidity has been emphasised in many studies (e.g. for Germany [5, 6]). International studies show that there are also strong inequalities with regard to LC, especially among men [7–16]. So far, however, little is known on how social inequalities in the incidence of LC developed over time. This holds especially true for Germany.

Few international studies investigated trends in social inequalities in LC morbidity and mortality. These studies mostly reported increasing social inequalities for men and women [11, 12, 15, 16]. Studies investigating social inequalities in the incidence of LC among the German population are rare. This is due to the fact that the data required for such analyses are scarce as official cancer registries do not include socioeconomic information of the diseased individuals. Current research from Germany based on cross-sectional data investigates whether social deprivation, measured by regional levels of income, education, and labour force participation, is associated with cancer. For these analyses, the macro-level information on regional social deprivation on district level was combined with cancer site-specific incidence rates. For LC, a social gradient was found in men but not in women [9]. However, as this solution is susceptible to ecological fallacies, the use of SES information on individual level should be preferred whenever possible. Furthermore, studies analysing time trends in SES inequalities in LC incidence in the German population are still lacking. Our study aims to step into this gap by using individual data on SES and incidence to examine time trends in inequalities in LC in Germany.

From a life course perspective, the cumulative disadvantaging effects of potentially harmful behaviours determine the outcome of social inequalities in health and mortality in later life [5, 17–19]. This holds especially for the harmful effects of smoking, as the risk of developing smoking-related diseases increases with the duration of smoking. The earlier individuals start smoking the more likely they are to suffer from a smoking-related disease later in life [20, 21]. Until the 1970s, smoking became more and more common but increasingly restrictive tobacco control policies in the 1990s and later had led to declining smoking rates, especially among highly educated individuals [17, 18, 21–26]. This development also had a positive impact on trends in mortality and life expectancy [27]. At the same time, smoking rates among individuals with low SES were quite stable or decreased at a slower pace than the rates among highly educated individuals [21, 26]. Moreover, a convergence of smoking rates between men and women has been observed in many industrialised countries since the 1960s and 1970s [18, 21, 25]. This convergence is rooted in changes in the social position of women and the adoption of risky health-related behaviours [18, 21, 25, 28, 29]. This has led to declining differences in smoking-related morbidity between men and women [13, 18, 21, 25, 28–32]. As a result, the burden of smoking-related diseases can be expected to concentrate in groups with lower SES. However, since the trends in smoking vary between sex and age groups, differing trends in social inequalities in LC incidence between men and women may also be expected [21, 33].

Due to data restrictions, many studies combined different indicators of SES (e.g. income, educational level, and occupation) into a single deprivation index. However, previous research has shown that these indicators measure different aspects of social inequality and should therefore be analysed independently whenever possible [34].

The aim of the study is to investigate time trends in LC incidence in Germany. Special attention will be paid to time trends of social inequalities in incidence and whether these trends differ between men and women, and between younger and older age groups. The analyses are based on claims data of a large German statutory health insurer, which contains large case numbers and different information on SES characteristics of the insured individuals.

The study is guided by the following research questions:

1. Are there socioeconomic inequalities in lung cancer incidence? Do these inequalities exist in men and women, and all age groups equally?
2. Are there different time trends in lung cancer incidence between socioeconomic groups? Are these trends in inequalities similar in men and in women?

## Methods

### Data

In this study, claims data of a large statutory health insurance provider (AOK Niedersachsen [AOKN]) were used, which insures approximately one third of the inhabitants of the federal state Lower Saxony [35]. The data of the years 2005 to 2017 were available for our analyses. As the number of incident cases of LC in the different SES groups was limited, single calendar years were sumarised into three time periods (2006–2009, 2010–2013 and 2014–2017). While it had been shown that the age and sex distribution of the insurance population is comparable to those of the total German population, individuals with low income and lower occupational position are overrepresented [36]. More detailed information on data characteristics can be found in previous studies [37–40]. The analyses were performed for all individuals aged 20 and older.

### Definition of lung cancer incidence

According to a previously published study based on the same data [39], cases of LC were identified based on the occurrence of an in- or outpatient LC ICD-10 diagnosis (C34.0 to C34.9) in the individual insurance history. Incident cases were defined for individuals having a LC diagnosis in the respective time period and who had a LC diagnosis-free period of at least 90 days preceding their initial diagnosis.

### Definition of socioeconomic indicators

Since insurance fees are based on the level of the individual income, the data contain information on the annual gross income from salaries and pension payments. Furthermore, information on educational level and occupational position is available for the employed population. Previous research has shown that each SES indicator depicts a different aspect of social inequalities and should thus not be used interchangeably due to their moderate or weak correlation [34, 41]. Therefore, each of these indicators was analysed separately to gain a deeper understanding of the underlying processes in SES inequalities in LC.

### Income groups

The income information contained in the data is based on the individual annual income reported to the insurer by the employer (working population) or by the Federal Pension Fund (retired population) [37–40]. Self-employed persons were also included in the analyses, as their insurance contributions also depend on their gross earnings [37–40]. As in previous studies, we defined income in relation to the German average income of a given year and adjusted it for inflation, which allows direct comparability over time as the purchasing power is kept constant [37–40]. Individuals were classified into three income groups according to their relative income level: Individuals with less than 60% of the German average income were assigned to the low, with 60 to 80% to the middle, and with more than 80% to the higher-income group [38]. The case number underlying the analyses of income inequalities in LC are presented in Table 1.

**Table 1:** Characteristics of the study population aged 20 and older: number of insured individuals, exposures in person-years, number of incident cases, and incidence per 100,000 by income group, time period, and gender

			2006–2009		2010–2013		2014–2017	
	**Income**		**Men**	**Women**	**Men**	**Women**	**Men**	**Women**
**working age (20–65)**	**low**	no. of individuals	232,254	290,532	251,755	341,196	271,634	375,622
		person-years	760,708	1,001,294	768,372	1,104,466	863,493	1,285,994
		no. of incident cases	1277	611	1197	781	1245	850
		incidence per 100,000	168	61	156	71	144	66
	**middle**	no. of individuals	72,486	58,126	97,185	70,631	113,485	89,995
		person-years	250,072	208,108	313,313	240,014	392,402	323,015
		no. of incident cases	192	94	242	105	252	135
		incidence per 100,000	77	45	77	43	64	42
	**higher**	no. of individuals	235,350	63,064	294,146	83,275	346,191	116,415
		person-years	848,419	228,704	1,015,157	288,553	1,252,295	414,364
		no. of incident cases	573	103	660	141	721	148
		incidence per 100,000	68	45	65	49	58	36
**retirement age (66+)**	**low**	no. of individuals	131,946	309,848	147,313	301,180	142,666	283,057
		person-years	465,746	1,114,705	506,319	1,062,912	497,975	1,011,342
		no. of incident cases	2957	1716	3183	1847	3189	2067
		incidence per 100,000	635	154	628	174	640	204
	**middle**	no. of individuals	66,519	39,639	63,839	40,211	65,993	46,095
		person-years	238,302	141,400	223,701	141,509	233,868	162,445
		no. of incident cases	1330	267	1166	282	1135	288
		incidence per 100,000	558	189	521	199	485	177
	**higher**	no. of individuals	18,310	12,635	17,545	13,268	23,468	17,277
		person-years	65,356	45,036	61,133	46,776	84,567	61,227
		no. of incident cases	347	101	266	101	306	125
		incidence per 100,000	531	224	435	216	362	204
**full age range (20+)**	**low**	no. of individuals	364,200	600,380	399,068	642,376	414,300	658,679
		person-years	1,226,453	2,115,999	1,274,691	2,167,378	1,361,468	2,297,336
		no. of incident cases	4234	2327	4380	2628	4434	2917
		incidence per 100,000	345	110	344	121	326	127
	**middle**	no. of individuals	139,005	97,765	161,024	110,842	179,478	136,090
		person-years	488,375	349,508	537,014	381,523	626,270	485,460
		no. of incident cases	1522	361	1408	387	1387	423
		incidence per 100,000	311	103	262	101	221	87
	**higher**	no. of individuals	253,660	75,699	311,691	96,543	369,659	133,692
		person-years	913,775	273,739	1,076,289	335,329	1,336,861	475,591
		no. of incident cases	920	204	926	242	1027	273
		incidence per 100,000	101	74	86	72	77	57

### Educational level

Educational level was defined using the years of schooling, which refer to different levels of school-leaving qualifications: 9 to 11 (low educational level), and 12 to 13 (high educational level) years of schooling. Since information on educational attainment is only available for the working-age population, the analyses of educational inequalities in LC incidence were limited to the age range 20 to 65 (Table 2).

**Table 2:** Characteristics of the study population aged 20–65: number of insured individuals, exposures in person-years, number of incident cases, and incidence per 100,000 by educational level, occupational group, time period, and gender

			2006–2009		2010–2013		2014–2017	
			Men	Women	Men	Women	Men	Women
**Educational** **level**	**low**	no. of individuals	410,758	304,628	486,722	351,816	509,839	375,616
		person-years	1,330,153	1,014,507	1,509,178	1,117,602	1,751,062	1,291,625
		no. of incident cases	845	335	1115	524	1357	665
		incidence per 100,000	64	33	74	47	78	51
	**high**	no. of individuals	55,354	61,794	77,235	86,732	95,232	110,026
		person-years	174,158	200,030	227,245	261,709	303,379	355,062
		no. of incident cases	24	12	53	30	95	59
		incidence per 100,000	14	6	23	11	31	17
**Occupational** **position**	**unskilled**	no. of individuals	214,665	141,850	199,094	144,150	171,384	138,432
		person-years	665,237	454,168	565,614	436,411	489,151	439,820
		no. of incident cases	571	237	506	282	330	302
		incidence per 100,000	86	52	89	65	67	69
	**skilled**	no. of individuals	167,548	64,081	267,444	101,476	350,375	149,766
		person-years	558,088	212,315	848,694	319,632	1,158,700	492,718
		no. of incident cases	260	44	494	100	769	175
		incidence per 100,000	47	21	58	31	66	36
	**specialist &** **highly qualified**	no. of individuals	59,285	108,140	92,970	147,217	122,805	187,899
		person-years	188,233	355,075	290,067	469,682	396,893	613,502
		no. of incident cases	66	98	136	163	148	199
		incidence per 100,000	35	28	47	35	37	32

### Occupational position

We defined occupational position according to the occupation classification system proposed by Blossfeld [42]. Within this system, individuals of the same occupational group are similar in terms of school-leaving qualification, vocational training, and professional activity. Due to the limited case numbers, we decided to combine the original 12 occupational subgroups into three groups: 1) unskilled, 2) skilled, and 3) specialists and highly qualified individuals. Individuals in the middle group usually have at least vocational training but no management function. In contrast, specialists and highly qualified individuals usually have a higher qualification (special professional training or university degree, e.g. bachelor or master level) and a higher level of decision latitude. The analyses concerning occupational inequalities in LC are also restricted to the working-age population (Table 2).

### Statistical analyses

Social inequalities in incidence risks were estimated using a two-stage approach, which has been well applied in previous studies (e.g. [37, 39, 40]). First, general inequalities in LC incidence risks among men and women were estimated by combining the data of the three periods and fitting parametric exponential proportional hazard regression models with constant baseline hazard over time. All analyses are controlled for the mean age in the period (as second-degree polynomial) and for period. To test the robustness of our models, we ran the same analyses with cox proportional hazard models, which, however, did not affect the results.

To analyse whether inequalities in LC incidence are age-patterned, we calculated the observed age-specific incidence rates and plotted them against the smoothed predicted incidence rates in a second step. The predicted rates were estimated from parametric proportional hazard models with an exponential distribution using the STATA command “predict” [43]. To examine whether inequalities increased or decreased over time we estimated interaction models (period*SES indicator).

All analyses were performed separately for the population at working age (20–65 years) and for the population at retirement age (66+ years) and for sex using Stata 14 [43]. All confidence intervals were estimated by drawing 1000 bootstrap samples.

## Results

### Social inequalities in lung cancer incidence

**Fig. 1:**
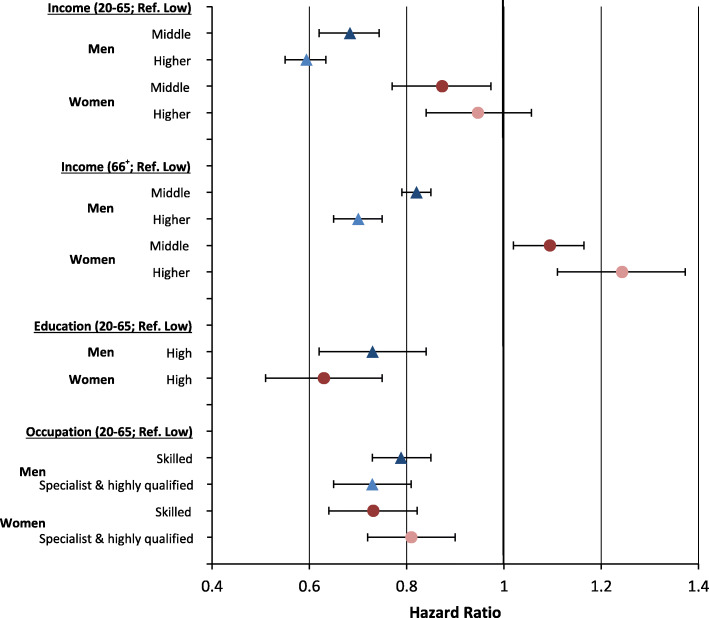
Risks of lung cancer incidence by socioeconomic group, stratified by sex and age. Note: all analyses are controlled for age (in single-year age-groups as second-degree polynomial) and for period.

With respect to income inequalities, a clear gradient in LC incidence emerged in men at working and retirement age (Fig. 1). Among women, the picture is less clear and the inequality patterns differ considerably between age-groups. While a tendency towards the typical income gradient can also be found among women of working age, the gradient turns at higher ages. Hence, the highest risks of LC incidence among women at retirement age were found for the higher-income group (HR = 1.2) (Fig. 1). With regard to educational inequalities, the analyses reveal that there are clear gradients in both sexes. Men and women with a high educational level have a 26 and 33% lower incidence risk than individuals with low educational level, respectively (Fig. 1). In men, the risk of developing LC decreases with the level of occupational qualification with the lowest risks among specialists and highly qualified men (HR = 0.7). Similar to men, the highest incidence risks were also found among women with the lowest level of occupational qualification (Fig. 1).

### Time trends in social inequalities in age-specific lung cancer incidence rates

**Fig. 2:**
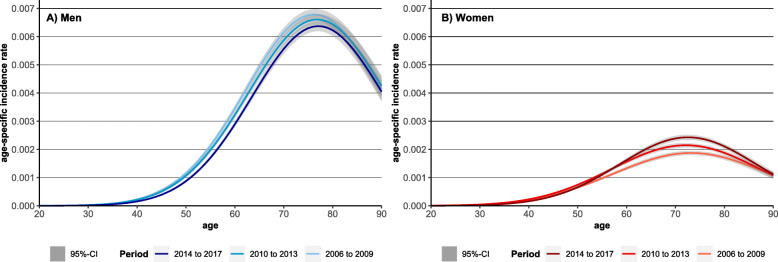
Observed and predicted values for age-specific lung cancer incidence rates for the periods 2006–2009 and 2014–2017 by sex

To analyse whether inequalities in LC incidence are age-patterned, age-specific incidence rates were analysed. Between the periods, decreasing age-specific incidence rates in LC were found among men, while the rates increased among women (Fig. 1). Furthermore, incidence rates among men shifted to higher ages which led to an increase in the age of incidence (Fig. 2).

**Fig. 3:**
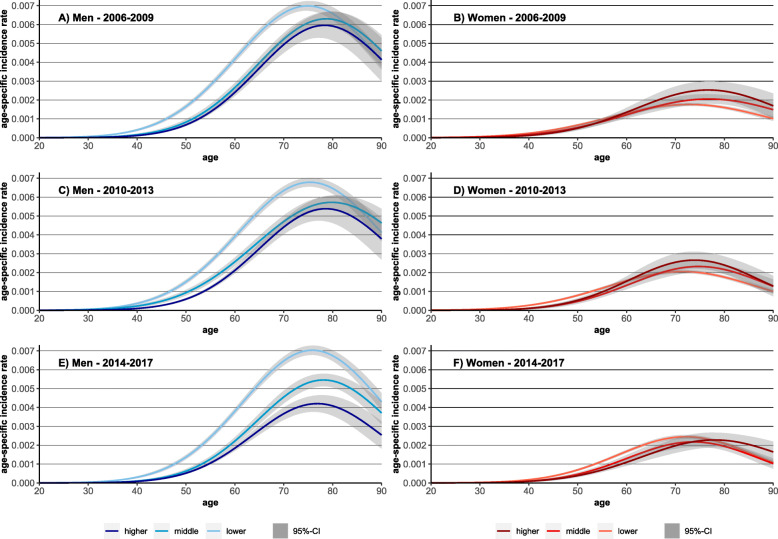
Observed and predicted values for lung cancer incidence rates for the periods 2006–2009 and 2014–2017 by sex and income groups

With respect to changes in income inequalities between the two periods, decreasing incidence rates in the middle- and higher-income groups among men were observed while the rates remained quite stable among men with low incomes (Figs. 1 and 3). A different trend was found in women. In the first period, differences in incidence rates between income groups were small for women at working-age. At older age, a reversed gradient emerged with the highest incidence rates among elderly women having higher incomes. Over time, the gradient turned. In the third period the highest incidence rates were found for women with low incomes at working age (Fig. 3). Accordingly, decreasing incidence risks in women with higher incomes and by decreasing incidence risks in women with low incomes were found (Fig. 1).

**Fig. 4:**
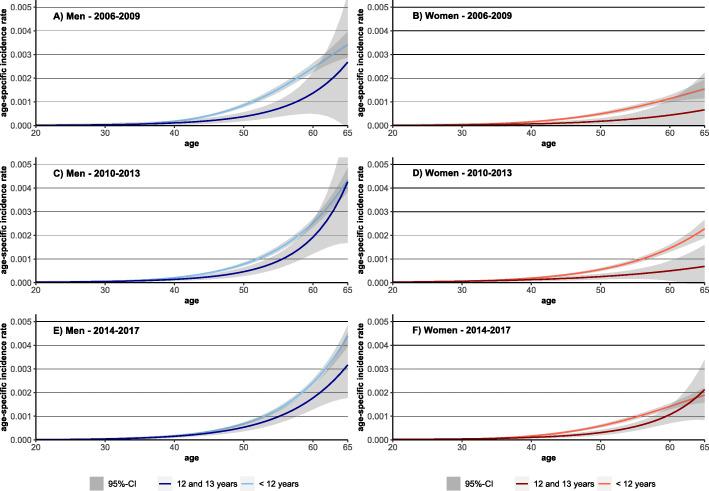
Observed and predicted values for age-specific lung cancer incidence rates for the periods 2006–2009 and 2014–2017 by sex and educational level

**Fig. 5:**
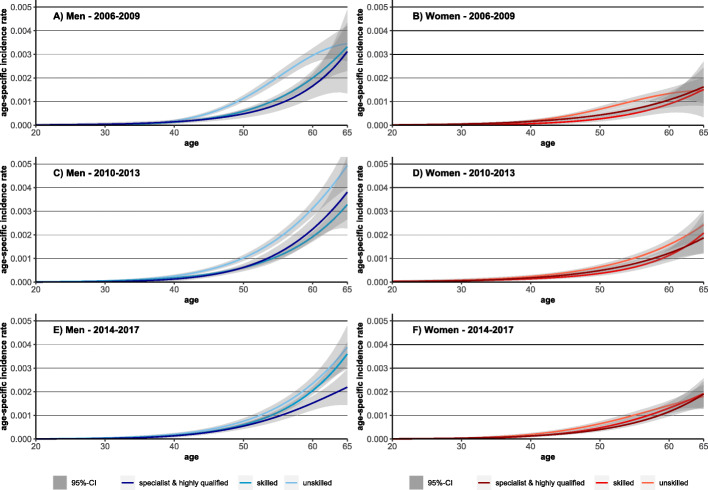
Observed and predicted values for age-specific lung cancer incidence rates for the periods 2006–2009 and 2014–2017 by sex and occupational position

Among individuals at working age, a slight increase in incidence rates among highly educated men led to decreasing differences between the educational groups over time. Similar trends were also observed among women (Figs. 1 and 4). With respect to occupational position, decreasing rates in men without vocational training (Fig. 1) led to a convergence between the different occupational groups (Fig. 5). In women, occupational inequalities are less pronounced than in men and tend to decrease over time as rates decreased slightly among women without vocational training (Fig. 5).

### Trends of social gradients in predicted lung cancer incidence rates

**Fig. 6:**
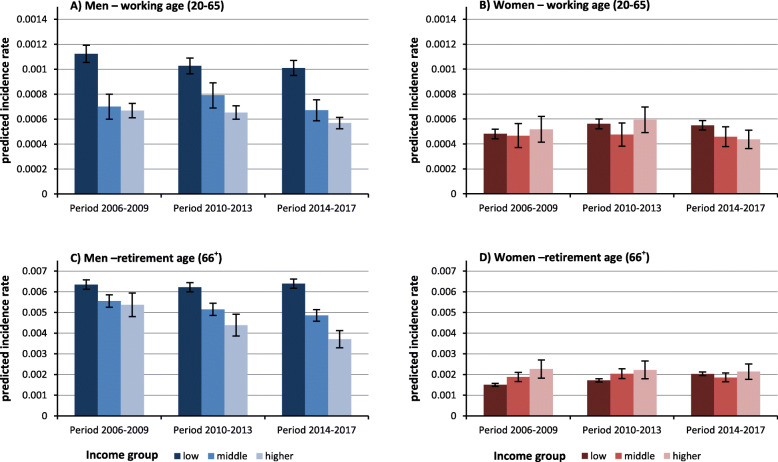
Predicted lung cancer incidence rate by income group, stratified for age and sex. Note: all analyses are controlled for mean age in period (as second-degree polynomial), for period; the models for the working-age population include all three socioeconomic indicators (income, occupational position, educational level) the models for the retirement-age population include income.

To identify whether inequalities narrowed or widened over time and which SES group accounted for these changes, we predicted LC incidence rates for different SES groups in the three time periods 2006–2009, 2010–2013, and 2014–2017 from interaction models (period*SES indicator). While income inequalities remained largely stable among men at working age, the gradient widened considerably among men at retirement age. This widening is mainly driven by the decline in rates in the middle and even more clearly in the higher-income group (Fig. 6). The reversed gradient observed in women at retirement age in the first period disappeared in the third period. Among women aged 20 to 65, the typical gradient emerged in the third period, indicating widening inequalities over time. In both subgroups, these developments are driven by the strong increase in incidence rates among women with low incomes and decreasing (age 20–65) or stable (age 66+) rates in the higher-income group (Fig. 6).

**Fig. 7:**
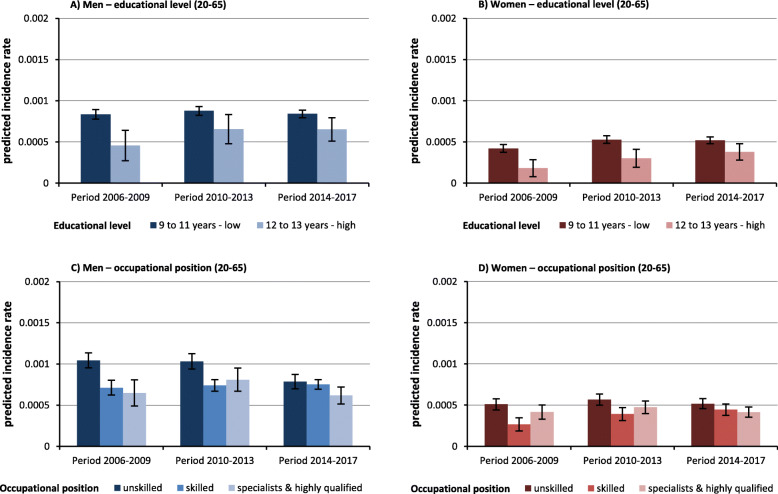
Predicted lung cancer incidence rate by education level and occupational position for the insurance population at working age, stratified for sex. Note: all analyses are controlled for mean age in period (as second-degree polynomial), for period; all models include the indicators for income, occupational position and educational level.

The educational inequalities in LC incidence among the working-age population narrowed over time. In particular, the tendency towards increasing rates among females and males with high educational level led to this converging trend between educational groups (Fig. 7). With respect to occupational groups narrowing inequalities in LC incidence were observed over time. In men, the strong decrease in the rate of men without vocational training led to this development. In women, on the other hand, it is the rising incidence rate among skilled women with vocational training that led to this trend (Fig. 7).

## Discussion

The aim of the study was to investigate time trends in socioeconomic inequalities in LC incidence in Germany. Our results concerning time trends in sex-specific incidence of LC are in line with the official statistics for Germany [2]. We found decreasing incidence rates in LC for men and increasing rates for women. This development had an impact on the gap between the sexes as the differences were reduced over time. In accordance with previous research [7–16], our findings reveal higher social disparities in LC for men than for women. These disparities were most pronounced in terms of income inequalities in LC incidence in men. Driven by the decline in LC incidence among the middle- and higher-income group, income inequalities widened in men. This increase in inequalities was strongest in men at retirement-age. In contrast, a reversed income gradient was found among women in the period 2006–2009. Due to rising LC incidence rates among women with low incomes and the decline in rates among women at working age with higher incomes, this gradient disappeared over time. Among men at working-age, the strongest inequalities in LC incidence were found in terms of income, the weakest in terms of occupational group, while among women, educational inequalities were strongest. Overall, occupational and educational inequalities tend to narrow over time.

Our study is one of the few that examines time trends in socioeconomic inequalities in LC incidence [11, 12]. International studies reported increasing inequalities in terms of social deprivation levels in LC morbidity for men and women for Great Britain [12, 16] and New Zealand [11]. Our findings are in line with current findings on LC incidence by regional deprivation level in Germany based on cross-sectional data, which reported substantial inequalities in men but not in women without considering differences in the working-age and retirement-age population [9]. In our analyses we may have indirectly depicted the results of changing regimes in harmful smoking behaviour in the 1960s and 1970s [18, 21, 25, 33], especially in women with higher SES, which is in accordance with a recently published study [39]. This underlines the importance of analysing time trends in SES inequalities in LC since cross-sectional approaches do not allow to depict the dynamics in cancer incidence within the different SES groups over time. This holds especially true for women. Thus, our findings may depict the effect of increased efforts in smoking prevention of the last decades [22, 24]. It should be noted that established tobacco prevention measures in Germany are weaker than in other European countries. Although progress has been made (e.g. smoking ban in public places), stricter anti-smoking measures are often called for but have not yet been implemented [4, 44]. However, our findings of the present and a recent study [39] indicate that 20 to 30 years later previous efforts in smoking prevention seem to have an impact on LC rates in women and men. Nevertheless, smoking-related diseases are still among the main driving forces of sex differences in mortality in Germany [32]. In contrast to the development at retirement age, inequalities among the working-age population are persisting or tend to narrow over time. This holds for educational as well as for occupational inequalities. With respect to occupational inequalities, this can most probably be explained by increasing standards of occupational safety, which among other things, resulted in lower exposure of carcinogens in the working environment over time. Among women at working-age, inequalities were strongest in terms of education while income inequalities in LC were much weaker than in men. This may be explained by the overall lower income level among women, which persists even when men and women with the same level of education are compared [45].

The findings indicate that trends in social inequalities in LC incidence may vary between age groups or birth cohorts. This holds especially true for women. However, as the majority of incident cases lies above the age of 70, the analyses concerning educational level and occupational positions are based on low numbers of incident cases. Therefore, it is important to continue to monitor developments to see whether trends in educational and occupational inequalities continue into old ages.

### Strengths and limitations

Our study is based on health insurance data spanning the time period from 2005 to 2017 that provide high case numbers and includes information on individual diagnoses as well as on socioeconomic characteristics. This permitted to analyse time trends in social inequalities in LC based on three SES indicators and among different age groups.

A major strength of our data is that all information is available at individual level, which prevents economic fallacies in the interpretation of the results [39]. Furthermore, the data contain the complete insurance population and are therefore not subject to selection bias with regard to health status [37–40, 46]. More detailed information on general strengths can be found in previous studies (e.g. [37–40]).

The precise information on individual income and high case numbers allow to obtain a detailed picture of the development in LC incidence in different income groups from age 20 to the oldest old as well as in the population subgroups at working and at retirement age. As for other studies based on health insurance data the results concerning income inequalities should be interpreted carefully since the data do not include information on household income (e.g. [37–40]). However, previous analyses have shown that social gradients in health obtained from estimates based on household income can largely be replicated using individual income, which suggests individual income to be an adequate measure to study social inequalities in health [47].

The data on educational graduation and occupational position are restricted to the population at working age. Due to this limitation, the analyses of trends in educational and occupational inequalities could not be conducted for individuals above age 65. It can be assumed that inequalities in LC caused by the former occupational position or the educational level persist into old age. Additional information on educational level and former occupation would have allowed a deeper insight into the developments of LC inequalities, especially among women, but cannot be analysed on the basis of our data.

As described in previous studies based on this data (e.g. [37–40], the data are representative for the total population of Germany in terms of sex and age structure but differ in terms of social distribution [36]. We addressed this limitation by stratifying or controlling all analyses for socioeconomic indicators. Therefore, the reported results should be unaffected.

## Conclusion

Our study reveals that social inequalities in LC are considerable and that trends vary with respect to SES group. The findings indicate that income inequalities widened among the elderly, but occupational and educational differences remained fairly stable or even narrowed among the working-age population. Most disadvantaged are men at retirement age with low income, for whom the increase in inequalities was most pronounced. More research is needed to uncover the underlying mechanisms that explain the widening inequalities in men and the observed trends in women. Our findings indicate that time trends in LC differ not only with respect to SES but also according to age range or birth cohort. The findings also suggest that focussing on social inequalities without considering differences between age-groups and time trends in health inequalities could lead to existing inequalities remaining undetected. In LC, this holds especially for the trajectories in inequalities among women over time.

It is important to foster public health interventions (e.g. complete ban of tobacco advertising and smoking in public places) to reduce LC incidence in the German population. Against the backdrop of existing inequalities, interventions should mainly focus on deprived social groups.

## Data Availability

The data analysed in this study are not publicly available due to protection of data privacy of the insured individuals by the AOK Niedersachsen (AOKN-Statutory Local Health Insurance of Lower Saxony). The data underlying this study belong to the AOKN. Interested researchers can send data access requests to Jona Stahmeyer at the AOKN using the following e-mail address: Jona.Stahmeyer@aok.nds.de. The authors did not have any special access privileges.

## Abbreviations

LC: lung cancer
SES: Socioeconomic status
AOKN: Allgemeine ortskrankenkasse niedersachsen
ICD-10: International classification of diseases 10th revision
HR: Hazard ratio
